# Enhancement of Light Amplification of CsPbBr_3_ Perovskite Quantum Dot Films via Surface Encapsulation by PMMA Polymer

**DOI:** 10.3390/polym13152574

**Published:** 2021-08-02

**Authors:** Saif M. H. Qaid, Hamid M. Ghaithan, Khulod K. AlHarbi, Bandar Ali Al-Asbahi, Abdullah S. Aldwayyan

**Affiliations:** 1 Physics and Astronomy Department, College of Science, King Saud University, Riyadh 11451, Saudi Arabia; hghaithan@ksu.edu.sa (H.M.G.); 438204190@student.ksu.edu.sa (K.K.A.); balasbahi@ksu.edu.sa (B.A.A.-A.); dwayyan@ksu.edu.sa (A.S.A.); 2 Department of Physics, Faculty of Science, Ibb University, Ibb 70270, Yemen; 3 Department of Physics, Faculty of Science, Sana’a University, Sana’a 12544, Yemen; 4 King Abdullah Institute for Nanotechnology, King Saud University, Riyadh 11451, Saudi Arabia; 5 K.A. CARE Energy Research and Innovation Center at Riyadh, Riyadh 11451, Saudi Arabia

**Keywords:** CsPbBr_3_ perovskite QDs, amplified spontaneous emission (ASE), light amplification, surface passivation, photostability

## Abstract

Photonic devices based on perovskite materials are considered promising alternatives for a wide range of these devices in the future because of their broad bandgaps and ability to contribute to light amplification. The current study investigates the possibility of improving the light amplification characteristics of CsPbBr_3_ perovskite quantum dot (PQD) films using the surface encapsulation technique. To further amplify emission within a perovskite layer, CsPbBr_3_ PQD films were sandwiched between two transparent layers of poly(methyl methacrylate) (PMMA) to create a highly flexible PMMA/PQD/PMMA waveguide film configuration. The prepared perovskite film, primed with a polymer layer coating, shows a marked improvement in both emission efficiency and amplified spontaneous emission (ASE)/laser threshold compared with bare perovskite films on glass substrates. Additionally, significantly improved photoluminescence (PL) and long decay lifetime were observed. Consequently, under pulse pumping in a picosecond duration, ASE with a reduction in ASE threshold of ~1.2 and 1.4 times the optical pumping threshold was observed for PQDs of films whose upper face was encapsulated and embedded within a cavity comprising two PMMA reflectors, respectively. Moreover, the exposure stability under laser pumping was greatly improved after adding the polymer coating to the top face of the perovskite film. Finally, this process improved the emission and PL in addition to enhancements in exposure stability. These results were ascribed in part to the passivation of defects in the perovskite top surface, accounting for the higher PL intensity, the slower PL relaxation, and for about 14 % of the ASE threshold decrease.

## 1. Introduction

Powerful and intense photoluminescence (PL), low non-radiative recombination rates, and long carrier lifetimes in pure and mixed perovskites have expanded their application in optoelectronic devices, such as light-emitting diodes (LEDs), lasers, and photodetectors [1,2,3,4,5,6,7,8,9]. Despite these features, controlling the morphology and thickness of perovskite films remain crucial to achieving high efficiency in a LED or in electricity production as defects and traps are essential to the movement of carriers in the material. Although inorganic cations (e.g., CsPbX_3_) show relatively improved stability compared with those of organic–inorganic hybrid counterparts (e.g., MAPbX_3_ and FAPbX_3_), CsPbX_3_ PQDs in practical operation are still very sensitive to polar solvents and moisture, anion exchange reactions, and heating. All of these are due to the low formation energy of the crystal lattice and the high decentralization activity of surface ions [2,3,4,5,6,7]. CsPbX_3_ nanocrystals (NCs) have received attention for their remarkable optoelectronic properties [8,9]. QDs, as well as other nanomaterials, have a large specific surface area, which greatly affects their intrinsic properties. Their inherent instability impedes further development and future application of CsPbX_3_ PQDs in optoelectronics and in other fields. Hence, it is crucial to explore an effective pathway to enhance the stability of CsPbX_3_ perovskite QDs.

To enhance the stability of PQDs, different protective strategies have been proposed by either modifying the surface ligand molecules of the PQDs [3,10,11,12,13,14,15,16] or by encapsulating PQDs into inorganic dielectric materials. Some examples of these strategies include ligand engineering, shell design, overcoating, and compositing PQDs with other materials. Composite materials, such as polymers, oxides, metallic ions, and other inorganics and organic options, passivate the PQD surface and form a protective layer [16,17,18]. All of these strategies and methods are practical and promising. For example, a surface modification strategy, by modifying ligand molecules, can passivate surface defects or dangling bonds to improve material stability. These encapsulation strategies can protect PQDs from exposure to corrosive exogenous species and enhance the stability of PQDs and other properties expected to improve performance in photonics, electronics, sensors, and other fields. The encapsulating can involve encapsulation by inorganic dielectric materials, for example, SiO_2_ [15,16,19,20], TiO_2_ [18,21,22], and organic poly(methyl methacrylate) (PMMA) polymer matrixes [23,24,25,26]. There are other methods of encapsulating using diverse structures, such as shelling the QDs at the single-particle level, encapsulating QDs in a broad matrix, loading QDs on a surface, ionic doping in the lattice of halide perovskite quantum dots, or forming halide perovskite QDs/QD nanocomposites. In addition to using these strategies to protect perovskites, the strategies contribute to the light amplification process. The light amplification and lasing properties of CsPbX_3_ PQDs can be enhanced through both high-quality surface passivation and high perovskite NC filling factors. There is feasibility of NC-based amplifiers and lasers tunable in the visible range because of their large energy gap (>1.75 eV), but these NCs cannot be used for light amplification in the infrared spectral range. Chemically synthesized NCs exhibit a wide range of size-controlled tunability of emission color and high PL quantum yields. These properties make NCs attractive materials for light-emitting applications ranging from bio-labeling and solid-state lighting to optical amplification and lasing [27].

The ASE in the CsPbX_3_ QDs were stimulated at ~7–15 μJ cm^−2^ and ~450 μJ cm^−2^ thresholds for excitation by femtosecond and nanosecond laser pulses, respectively [28]. Additionally, the ASE thresholds of CsPbBr_3_ thin films under femtosecond laser system were reported and two-photons using a femtosecond laser system as pumping lasers were 192 μJ cm^–2^ and 12 mJ cm^−2^, respectively [28,29]. Another group studies the role of the ligand in improvements of stability problem of CsPbBr_3_ QDs in air [14]. The ASE thresholds of CsPbBr_3_ thin films were demonstrated under picosecond laser excitation around 22.5 μJ cm^−2^, which confirmed the role of laser pulse duration in determined the ASE threshold [30]. In one of the previously published works, the ASE threshold in CsPbBr_3_ quantum dot films was reducing through controlling the TiO_2_ compact layer under perovskite film by the reduced roughness of the obtained films to less than 5 nm with 50 nm TiO_2_ substrate [31].

In view of these reports, which listed different ways to improve the properties of ASE, the accelerated research activity has allowed many groups interested in studying perovskite materials and their applications in light-emitting, to propose approaches to improve the ASE and lasing properties that address a number of aspects, such as reducing the threshold of ASE/laser, laser cavity geometry, wavelength range engineering, and stability improvement. However, the inherent instability of perovskite materials hampers further development and future application of these materials in optoelectronics and in other fields.

Herein, a simple strategy will be demonstrated for improving optical properties by modifying the upper and lower surfaces of a CsPbBr_3_ film by coating it with PMMA polymer, which leads to improving the perovskites materials stability and light amplification at the same time. Then, the basic physics of light amplification in the CsPbBr_3_ PQD with emission energies in the visible range was analyzed. For this reason, high-quality films were fabricated directly from CsPbBr_3_ PQD in powder form. The top face of the perovskite layer was coated by PMMA, and the CsPbBr_3_ PQD films were placed between two transparent PMMA layers. These simple methods result in the formation of a highly flexible ultra-light PMMA/PQD/PMMA waveguide film configuration used to examine the optical response of perovskite films after PMMA surface passivation and waveguide fabrication.

## 2. Materials and Methods

### 2.1. Materials

Cesium lead bromide quantum dot powders was purchased from Quantum Solutions Company (Thuwal, Saudi Arabia, www.qdot.inc (accessed on January 2021)). N-Hexane analytical reagent solution was purchased from (Avonchem, Cheshire, UK). Poly(methyl methacrylate) (PMMA) with an average molecular weight of ~120,000 g/mol was purchased from Sigma-Aldrich (Saint Louis, MO, USA). All chemicals were used as received, without further purification.

#### 2.1.1. Fabrication of CsPbBr_3_ QD Solution and Thin Films

The powdered CsPbBr_3_ PQDs were directly dispersed into hexane (25 mg/mL) for suspension. Then, the suspension was left overnight to ensure complete dispersion before thin film fabrication. To fabricate thin films, CsPbBr_3_ PQDs were coated onto pre-cleaned microscope glass (1 × 2 cm^2^) substrates. The PQD mixture (50 µL/cm^2^) was dropped onto the substrate and spin-coated at 4000 rpm for 30 s. Then, the films were dried under vacuum for 1 h. The CsPbBr_3_ PQDs film thickness could be adjusting to 300 nm (estimated from a Dektak 150 stylus profiler (Bruker Corp, Tucson, AZ, USA)) in all configurations to compare the difference on ASE performance.

#### 2.1.2. Preparation of PMMA Solution and Modification of the Perovskite Surface for Encapsulation

**Figure 1:**
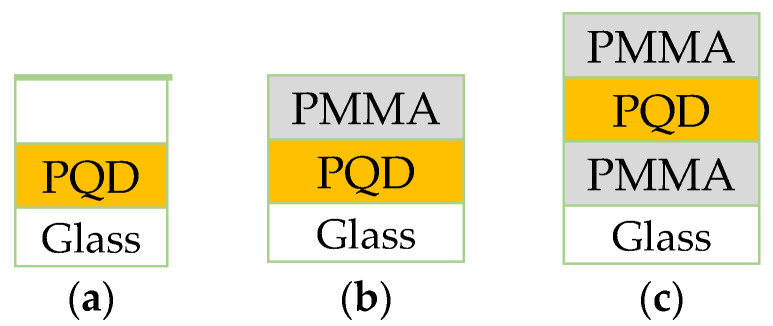
Configuration schematic of the (**a**) PQD; (**b**) PMMA/PQD; (**c**) PMMA/PQD/PMMA; All configurations fabricated on glass substrates.

First, the PMMA stock solution was prepared in toluene (25 mg/mL). For the encapsulation of PQDs, PMMA thin film was prepared by depositing 25 µL/cm^2^ of the PMMA solution onto PQD films using a spin-coating procedure (6500 rpm for 30 s) under ambient conditions. Next, the PMMA/CsPbBr_3_ PQD films were dried in ambient air also. Finally, for PMMA thickness measurements, pure PMMA films were condensed from the stock solution onto a clean glass substrate by the spin-coating procedure. The film thickness could be adjusting to the required PMMA layer thickness (100 nm). After that section, the thickness will be referred to the PQD structure only. Figure 1 shows a schematic for all configurations CsPbBr_3_/glass, PMMA/CsPbBr_3_/glass, and PMMA/CsPbBr_3_/PMMA/glass, respectively.

### 2.2. Characterization

Structural characterization: Quantum dot structure and morphology of the CsPbBr_3_ perovskite were analyzed via transmission electron microscopy (TEM; JEOL JEM-1011, JEOL, Tokyo, Japan). The samples were prepared by adding a few drops of dilute PQD solution onto TEM grids. The crystallization structures and the crystal phase of CsPbBr_3_ PQDs were characterized using X-ray diffraction (XRD) analysis (Miniflex 600 XRD, Rigaku, Japan) with a copper Kα radiation source (λ = 1.5418 Å). The scanning range was 2θ = 10°–80° for a scan rate of 3° min^−1^ with a step size of 0.02°.

#### 2.2.1. Optical Characterization

**Figure 2:**
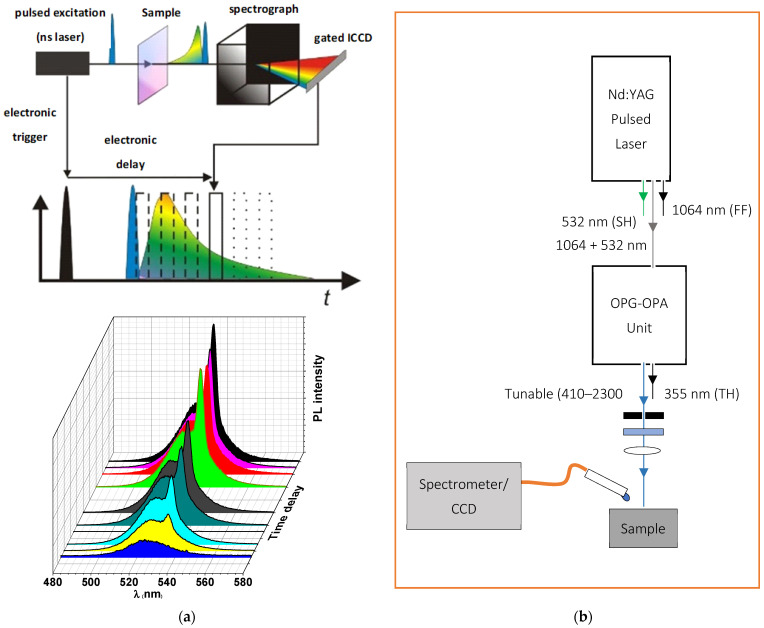
(**a**) The upper part shows the schematic of the ICCD setup with the beam path and the shifted integration window compared to the excitation window. The lower graph shows the building up of the measured signal over several single measurements; (**b**) Schematic showing the High Power Picosecond Pulsed Time Integrated PL Setup for characterizing the presence of stimulated emission in thin-film configuration.

Absorption and photoluminescence (PL) measurements of the PQD thin films were recorded in the 350–700 nm spectral range using a V-670 UV-vis spectrophotometer (JASCO Corp., Tokyo, Japan) and a fluorescence spectrophotometer (Lumina, Thermo Fisher Scientific, Madison, WI, USA), respectively. In both measurements, a portion of the CsPbBr_3_ PQD suspension was dispersed onto a microscopic slide with a thickness of approximately 300 nm. The resulting films were checked via observation by naked eye under a UV lamp (model XX15NF, Spectroline, ME, USA) at 365 nm. Furthermore, steady-state measurements and time-resolved PL (TRPL) were performed using a Shamrock SR-500i spectrometer (Andor Technology Co, Belfast, UK) equipped with an MS257 ICCD detector (Lot Oriel Instruments, Stratford, CT, USA). For sample excitation, a pulsed laser was used via the third harmonic generation of a Q-switched Nd: YAG nanosecond laser (Solara, LPS 1500, 3rd harmonic, wavelength: 355 nm, pulse width: 11 ns, repetition rate: 100 Hz, energy density: 1.5 μJ cm^−2^). To collect the laser excitation pulse from the detector and select the wavelength emitted from the sample, special filters will be used for this purpose. Moreover, it will be use a lens to collect and focus light emitted from the samples. The resulting emission is spectrally resolved using a spectrograph and detected by a gated intensified and a sufficiently sensitive ICCD camera. By a sequential shift of the gate window (to change time delay over a range from 1 ns up to 1 ms) with respect to the excitation, it is possible to measure the spectrally resolved decay of the photoluminescence, providing information about the excited state. A schematic diagram of the ICCD setup experimental used to measure the TRPL is shown in Figure 2a.

#### 2.2.2. Laser Experiments and ASE Measurements

To investigate the ASE characteristics, energy-dependent ASE intensity spectra were collected at the sample edges, in particular near the ends of the excitation strips. A LOTUS II Q-switched Nd:YAG picosecond laser (LOTIS, Belarus) with a pulse duration of 70–80 ps at a repetition rate of 15 Hz was used for excitation while using an LT-2215-OPG optical parametric generator (OPG) with a tunable range of 425–2300 nm. Then, a cylindrical lens was used as a focusing tool to create a narrow excitation stripe with a 100 µm width of variable length on the sample surface to be sure that the collection efficiency and intensity profile were effectively constant across the lengths of the stripe used. The light emitted by the samples was detected from the edge of the waveguides by using an optical fiber connected to A QE65 Pro spectrograph (Ocean Optics, Inc., Dunedin, FL, USA). To enable the study of the threshold dependence on energy density, the laser energy density was attenuated using a variable neutral density filter wheel and the energy was read by an LM-P-209 coherent thermal sensor head. Finally, to analyze the data collected from ASE experiments and to obtain gaussian fits of dual PL and ASE emission peaks, a custom python-based program, developed by our research group, was used. A scheme diagram of the laser setup experimental used to investigate the presence of stimulated emission is shown in Figure 2b.

## 3. Results

### 3.1. Structural Characteristics

**Figure 3:**
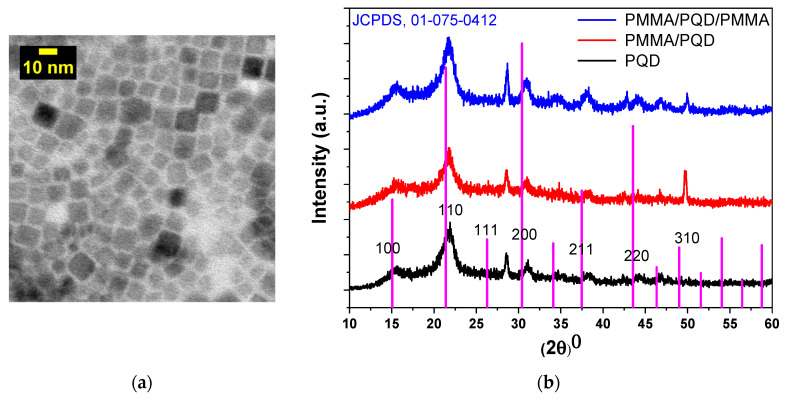
(**a**) TEM image of CsPbBr_3_ PQDs; the CsPbBr_3_ QDs sample for TEM investigation was prepared by the dilution of CsPbBr_3_ QDs solution to (125 µg/1 mL), followed by placing several drops on a carbon-coated copper grid (**b**) X-ray diffraction patterns of CsPbBr_3_ PQDs films for all configurations fabricated on glass substrates.

The TEM image in Figure 3a shows the structure of the CsPbBr_3_ PQDs material and reveals that the CsPbBr_3_ PQDs have a uniform shape and homogeneous size distribution. The particle sizes range from ~4 to 11 nm with an average particle size of ~7.5 nm. Figure 3b shows the XRD patterns of perovskite films with and without PMMA polymer coating. The XRD patterns have characteristic peaks at (2*θ* = 15.54°, 21.90°, 31.09°, and 51.56°), which correspond to diffraction from (100), (110), (200), and (311) crystal planes, respectively. All peaks were indexed to cubic phase in the Pm-3m space group (221) and XRD pattern samples could be indexed to the pure cubic phase of CsPbBr_3_ (JCPDS card no. 01-075-0412), with slight peak shifts. Slight shifts in peak positions (~0.6°) were consistent with alloy formation and the results are well in line with previous reports [31]. The peak shifts decrease when the top surface of the PQD was modified by the PMMA polymer; they revert to appear as in the bare surface of PQD when the PQD top and bottom surface are modified. Although, the XRD peak at 51.56° was apparent from pristine-CsPbBr_3_ PQDs and was not apparent from polymer due to the polymers did not readily absorb in the X-ray region [26,32,33], as evidenced by the XRD patterns of the experimental samples. The strong peak after adding the polymer may be attributed to the film quality improvement. Diffraction from the (200) plane was apparent, along with the secondary diffraction peak of the (100) plane, indicating the existence of a very pure and crystalline cubic phase, without any defects. The appearance of the peak at 28.56° in pristine-CsPbBr_3_ PQDs exhibited a mixture of predominant cubic phase and a minor portion of the orthorhombic phase, which maybe be attributed to the stored age of PQDs under ambient conditions in this experiment is 6 months from production [34].

The Scherrer formula ($D=0.9\frac{\lambda }{\beta \cos\theta }$) was used to estimate the crystallite size (D). Additionally, the dislocation density (δ) and lattice strain (ε) are given by $\delta =\frac{1}{D^{2}}$ and ε= βcosθ/4, respectively [30,35]. β represents peak broadening (FWHM), λ is the wavelength of the incident X-ray (0.154 nm), and k is a constant (~0.9). Table 1 lists the values of XRD parameters.

The crystallite sizes were estimated from the Scherrer formula to be 5.8, 6.6, and 6 nm for pure PQDs, PMMA/PQDs, and PMMA/PQDs/PMMA, respectively. Compared with the TEM image, the XRD results were consistent and broadly in agreement. The gradual formation of the perovskite layer reduced the strain. The narrow linewidth of the diffraction peaks in the XRD patterns (FWHMs) indicated the lowering of residual stress in the crystals and a low dislocation density; generally, a high-quality perovskite film with a low density of defect states [35]. Since PMMA is a highly transparent amorphous polymer, it does not exhibit any sharp diffraction peaks in the XRD spectra due to not readily absorb in the X-ray region [36]. The agglomeration of PQDs on the polymer segments is the reason for the observed increase in grain size. The sandwiched PQD by PMMA shows a lower grain size than PMMA/PQD. The reason behind this may be due to the confined of the QDs between the PMMA layer. Additionally, grain growth rates increase in the film because of stresses in the film, which are created within the film and substrate and usually with no dislocations at the interface.

### 3.2. UV-Vis Absorption and Steady-State Photoluminescence Properties

**Figure 4:**
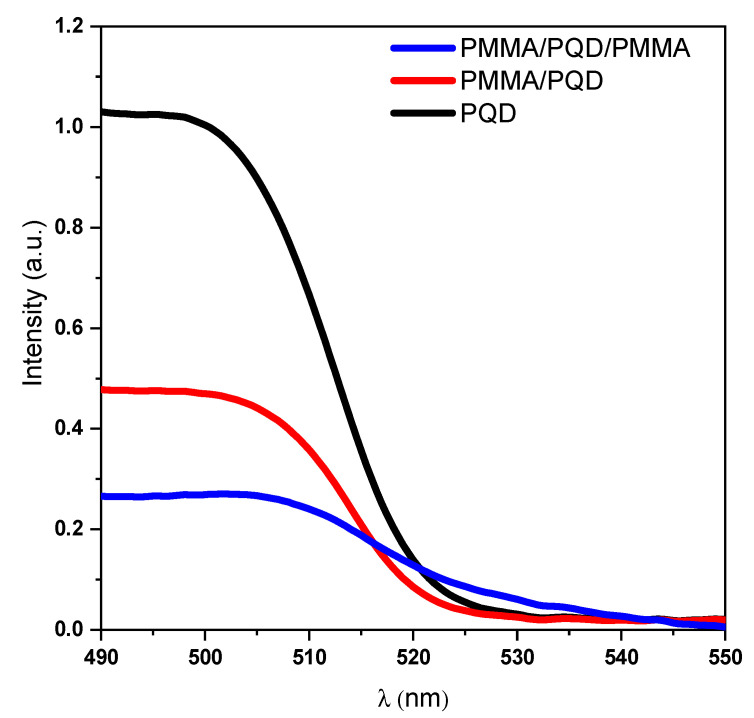
Optical absorption spectra of CsPbBr_3_ PQDs films at room temperature.

**Figure 5:**
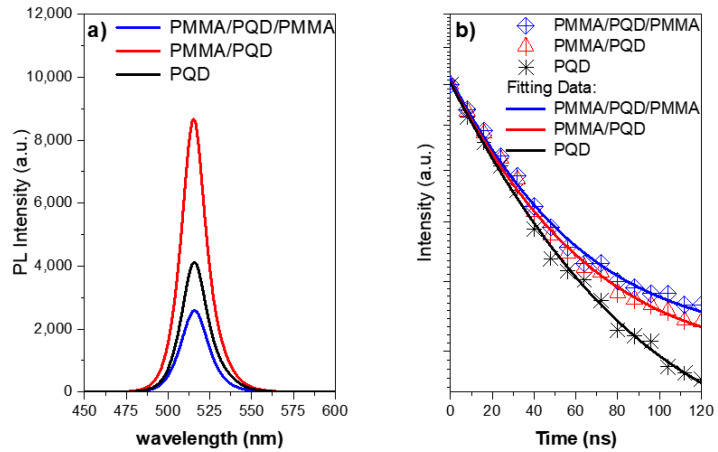
(**a**) Steady-state PL spectra of CsPbBr_3_ PQD films measured at room temperature under 410 nm excitation. The inset shows a real-color image CsPbBr_3_ PQD excited under 365 nm UV lamp; (**b**) TRPL decay plots of CsPbBr_3_ PQD thin films at low pump energy densities.

Figure 4 and Figure 5a show the UV-vis absorption and steady-state photoluminescence (PL) spectra of the bare perovskite film and that modified by PMMA polymer in one and two faces. These results correspond well to perovskite film results reported for CsPbBr_3_ films, with only a change in intensity [30,31]. Additionally, the high crystallinity of the perovskite film was confirmed by the observation of narrow-band emission (FWHM) located at 516 nm, which indicates a low density of defect states as shown in XRD results.

Thus, the high-quality of the perovskite film came from the smoothing of the surface of the film after covering it with a polymer layer, which is evident from the increase in the emission intensity. After covering the top of the perovskite film with a polymer layer, the PL intensity increased at the same pump fluence compared with that of the bare film (Figure 5a). This is expected due to the fact that the polymer layer improves the interface and thus the smooth surface reduces the loss of pumping light incident at the air interface with the PQD thin film [31,33,37]. Although this increase is reduced when the perovskite film is sandwich between two polymer layers. The role of the layers that sandwiched the perovskite films plays is to redirect the emission to propagate along the path inside the perovskite to go out from the edge. This behavior is attributed to the steady-state photoluminescence (PL) measurements taken at a 45° angle in the PL steady-state. Therefore, the losses of the output light from the PMMA/PQD/PMMA sample will be much higher than the other samples. Whereas the emission is guided by the two PMMA polymer layers; this high emission taken from the edge will be discussed later in the ASE studies. The inset of Figure 5a shows a photograph of the film taken under UV light (*λ_ex_* = 365 nm).

Additionally, to further understand the effect of the surface passivation layer in the perovskite top surface, time-resolved PL (TRPL) studies were conducted when created photo-generated carriers after the laser excitation pulses as can be illustrated in the characterization section and shown in Figure 5b. The PL lifetimes and decay component can be measured from studies of the emission intensity with decay time, which can be deduced from the PL decay curve. The PL decay profile was fitted at the peak position using single-exponential decay with *I* (*t*) = *A* exp (−*t*/*τ*), where *τ* is the average lifetime. The PL decay curve with a component, shown in Figure 5b, revealed an emission with a PMMA/PQD/PMMA and PMMA/PQD decay time of ~17.0 ns and 16.4, respectively, which is longer than that of the PQD (~14.0 ns), which arose because of surface passivation which the passivation of defects in the top of perovskite surface [37,38]. So, the average lifetime increased with surface passivation and the passivation surface have a lower surface recombination velocity than that obtained from the bare surface. Moreover, the intensity of the coated film was considerably higher than that caused by the bare film due to slow PL relaxation dynamics (long decay time). These results were ascribed in part to the passivation of defects in the perovskite top surface.

### 3.3. Light Amplification and ASE Properties

**Figure 6:**
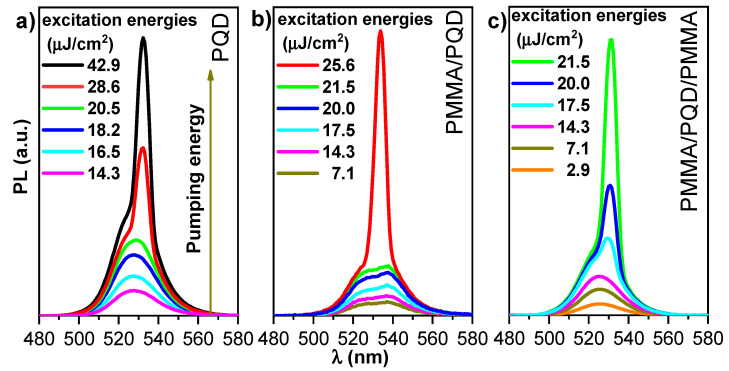
PL spectra measured at room temperature versus excitation energy density for (**a**) bare CsPbBr_3_ PQDs; (**b**) PMMA/PQDs, and (**c**) PMMA/PQDs/PMMA with pulsed excitation (410 nm, 70 ps pulses, 15 Hz repetition rate).

Evaluation of photoluminescence with optical pumping: As demonstrated in previously published work [23], the threshold properties and gain characteristics of PMMA/perovskite can be controlled by changing the polymer thickness. Here, Figure 6 shows the dependence of the excitation energy density of PL spectra in a wide energy range (low and high excitation energy). This figure shows the pump-dependent emission from three configurations, CsPbBr_3_/glass, PMMA/CsPbBr_3_/glass, and PMMA/CsPbBr_3_/PMMA/glass, through a range of excitation energy densities at room temperature (T = 300 K).

**Figure 7:**
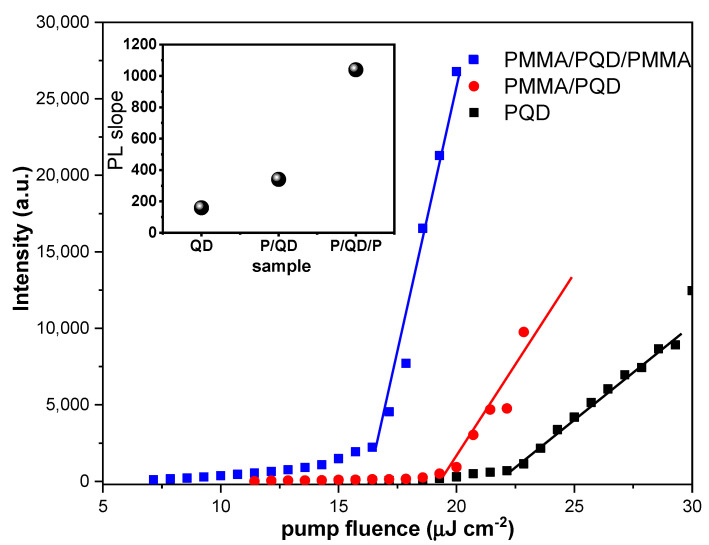
Output light-input light curve of the emission spectra versus excitation pump energy. The inset shows the quantum efficiency behavior.

In the CsPbBr_3_/glass configuration, the transition from a broad PL spectrum to a narrower ASE feature, with the appearance of a narrow band peaked at 535 nm, occurred at 22.2 μJ/cm^2^. By contrast, the configurations PMMA/CsPbBr_3_ and PMMA/CsPbBr_3_/PMMA show broad PL, even at the lowest excitation energy density, at 19.3 and 16.4 μJ/cm^2^, respectively. Thus, the presented results confirmed that the ASE feature was observed for all configurations, but the use of a PMMA passivation layer consistently yielded stronger ASE density, lower ASE thresholds, and high emission control. At low excitation energy density, the PL peak at 525 nm has an FWHM of ~16.5 nm. Line–shape variation can be observed as the excitation density increases. Increasing the excitation energy density results in a transition from a broad PL spectrum to a narrower ASE feature with a narrow band peaked at 535 nm. At low pumping energy, the PL appeared at a broad peak, but when pumping energy increase until the pump energy reach threshold, the sharp peak appeared near the long wavelength. The broad peak dispersed at the ASE became dominant in this state. However, when the pump energy increases above the ASE threshold, a redshifted peak has multiple causes, such as defect transitions, thermal effects [39], and re-absorption effect arising from the overlap of the absorption band edge with the PL emission (spontaneous emission spectrum) (Figure 4), the self-absorption effect should contribute to the ASE state [40]. Moreover, as suggested by band gap renormalization in the highly excited perovskite crystal [41], the band gap is redshifted by hole–electron interactions under high population conditions. The peak transition from a broad PL spectrum to a narrower ASE feature also reflects the bandgap behavior. The wavelength of the PL peak shows a progressive red shift (~10 nm) up to the maximum investigated excitation energy density. Then, the emission changes from broad PL (FWHM of ~17 nm) at low fluence to ASE (FWHM of ~6 nm) at high fluence. The transition from a broad PL spectrum to a narrower ASE feature occurs at a threshold fluence of ~16–23 μJ/cm^2^. The visible excitation energy density, estimated by determining the mean value of the minimum pump energy density that allows observation of the ASE regime, was measured in three different positions on the sample and was approximately 22.2, 19.3, and 16.4 μJ cm^−2^ for the CsPbBr_3_/glass, PMMA/CsPbBr_3_/glass, and PMMA/CsPbBr_3_/PMMA configurations, respectively. The ASE threshold was estimated from PL excitation density dependence analysis by fitting the low excitation density data point and the high excitation density portion of the data with a constant and an increasing straight line, respectively, while considering the threshold crossing point between the two fitting lines (Figure 7). Additionally, the PL intensity of the coating film was stronger than the intensity of the bare film, as can be seen from Figure 7. This mechanism is often referred to as a smoother surface after covering the PMMA polymer layer, the passivation of defects in the perovskite top surface, and as index guiding since the refractive index discontinuity between the active (CsPbBr_3_ PQD) and cladding (PMMA) layers is responsible for mode confinement through total internal reflection occurring at the interface. Here, the refractive index of cladding is almost identical to that of the glass substrate (n = 1.5) and smaller than that of active layer (n ≈ 2); at λ= 410 nm [42,43,44]. The inset of Figure 7 shows that the PMMA/CsPbBr_3_/PMMA configuration has a higher quantum efficiency (as can be deduced from its larger slope in the linear region). Indeed, the strong contrast between the three configurations clearly illustrates the importance of the enhancement of the film surface and light guiding in the optical and gain characteristics of the perovskite films. The PMMA-coated film showed slower PL relaxation dynamics (long decay time). These results were ascribed in part to the passivation of defects in the top of perovskite surface, accounting for the higher PL intensity, the slower PL relaxation and for about 14 % of the ASE threshold decrease. The remaining ASE threshold decrease was instead ascribed to improved waveguiding, which was facilitated by the realization that an almost symmetric glass-perovskite-PMMA waveguide results in higher mode confinement in the perovskite layer concerning the asymmetric glass–perovskite layer. This suggests that the PMMA layer redirects emission that propagates along the out-of-plane and oblique paths back into the bulk. Consequently, the optical path length in the medium is increased, resulting in a lower ASE threshold compared with the bare PQDs. Here, the PMMA layer is effectively improving the waveguiding capability of the perovskite film, these findings compatible with the previous studies in the open literature [23,31,37,38,45,46]. Additionally, in all of these, the ASE threshold could further reduce with one or both reflectivity/encapsulation substrates.

**Figure 8:**
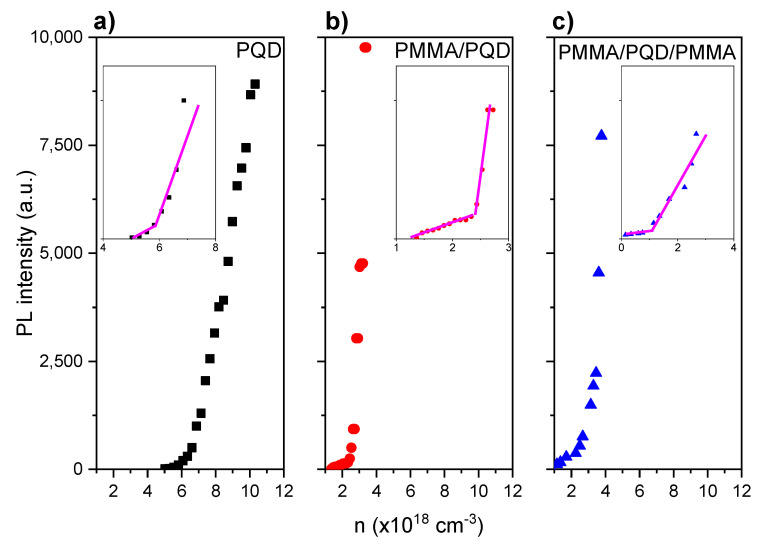
Integrated PL versus carrier density, n. (**a**) bare CsPbBr_3_ PQDs, (**b**) PMMA/PQDs, and (**c**) PMMA/PQDs/PMMA films deposited on the microscopic glass.

Figure 8 depicts the ASE intensity dependence as a function of carrier density (*n*). The carrier densities are calculated from the absorbance spectra (Figure 4) and film thickness (estimated from a Dektak 150 stylus profiler). The calculation method has been explained in detail in previously published work [31,47]. The transition from spontaneous emission (SE) to ASE occurs, with a clear onset, at ~1.8–6.7 × 10^18^ cm^−3^ as shown from spectra that were magnified at the onset of ASE as shown as in the inset of Figure 8. The carrier density threshold was estimated from PL excitation density dependence analysis by fitting the low excitation density data point and the high excitation density portion of the data with a constant and an increasing straight line, respectively, while considering the threshold crossing point between the two fitting lines. From Figure 8, CsPbBr_3_/glass shows a sharp SE to ASE transition at a pump fluence of 22.2 μJ/cm^2^. This fluence value corresponds to an ASE threshold carrier density of ~6.7 × 10^18^ cm^−3^. This sample showed slower ASE growth with increasing pump fluence. A sharp SE to ASE transition for PMMA/CsPbBr_3_/glass and PMMA/CsPbBr_3_/PMMA/glass appeared at a pump fluence of 19.3 and 16.4 μJ cm^−2^, respectively. These threshold values correspond to a threshold carrier density of ~2.8 × 10^18^ cm^−3^ and 1.8 × 10^18^ cm^−3^, respectively. Thus, the PMMA/CsPbBr_3_/PMMA/glass configuration is the best for gain and light amplification applications. This is because, for very high carrier densities, the Coulomb interaction between electrons and holes can induce additional effects. Auger recombination is one effect that can severely reduce the gain. The bandgap energy and exciton binding energy are both pushed to transfer to the lower values at carrier density pushed to higher values. This process is called renormalization, whereas when all excitons in the electron hole plasma are ionized at the threshold point of carrier density, a “Mott transition” occurs.

**Figure 9:**
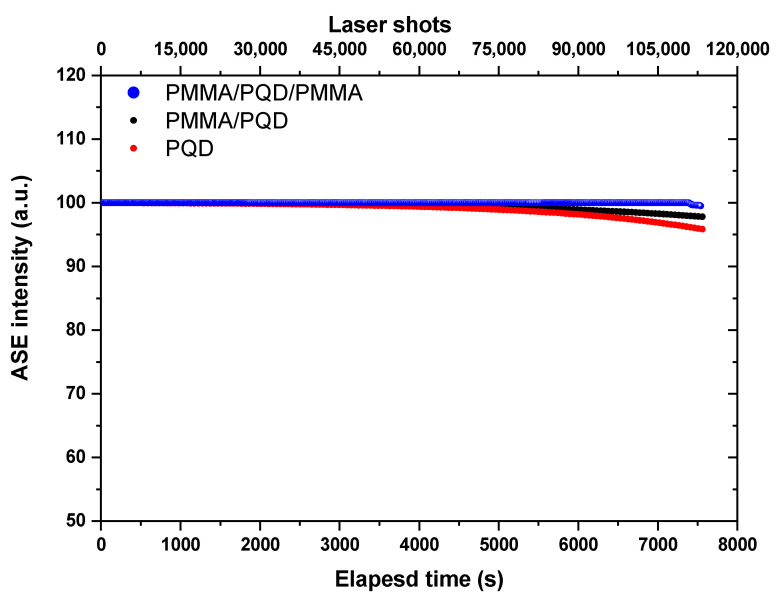
ASE intensity for CsPbBr_3_ PQDs stability studies under a constant pulsed excitation density of 40 μJ/cm^2^ for over 120 min in an ambient atmosphere.

Photostability studies: The time-dependent ASE intensity of synthesized films, before and after PMMA coating, with over 120 min laser excitation lasts without interruption (for 108,000 excitation shots). Figure 9 shows the ASE stability under long excitation by picosecond laser pulses (pulse width is ≈70 ps and repetition rate is 15 Hz). For an excitation wavelength of 410 nm, the excitation energy is adjusted above the threshold (~twice the energy threshold) with a run at room temperature and under atmospheric conditions. It is noted that, for the film coated by PMMA, the intensity output does not deviate from the original output until more than 110,000 excitation shots. By contrast, the ASE, even after 80,000 shots, from the bare sample without coating, can remain at approximately 95% of its original emission intensity. Thus, the ASE photostability of synthesized films is significantly improved after PMMA coating. The improved stability can be ascribed to ligands engineering (lengths of ligands—the short branched chains) of the CsPbBr_3_ PQD [48,49] and also by incorporation into hydrophobic polymer matrices [45]. The short branched chains will be increase the binding energy between the ligands and QDs and the lengths of ligands, which is related to the strength of the van der Waals (VDW) interactions among the ligands, and the strength is dominant to determine the crystalline structure and follow the optical properties of PQDs [49,50,51,52].

## 4. Discussion

At the beginning, the TEM image and XRD patterns for three configurations, CsPbBr_3_/glass, PMMA/CsPbBr_3_/glass, and PMMA/CsPbBr_3_/PMMA/glass (Figure 3a,b) confirm that the grown of CsPbBr_3_ PQD films are of high phase purity and the PQD have a uniform shape and homogeneous size distribution. All XRD peaks were indexed to cubic phase in the Pm-3m space group (221) and XRD pattern samples could be indexed to the predominant cubic phase with slight peak shifts, which are consistent with alloy formation [30,31]. The peak shifts decrease when the top surface of the PQD was modified by the PMMA polymer; they revert to appear as in the bare surface of PQD when the PQD top and bottom surface are modified. Although the PMMA polymer is highly transparent amorphous and do not readily absorb in the X-ray region, the strong peak after adding the polymer may be attributed to the film quality improvement. The UV-vis absorption results (Figure 4) correspond well to perovskite film without any effect for polymer the change the CsPbBr_3_ PQD band gap, with only a change in intensity [30,31].

The steady-state photoluminescence (PL) spectra of the bare perovskite film and that modified by PMMA polymer in one and two faces also was investigated. From the PL investigation, the PL intensity increased at the same pump fluence after covering the top of the perovskite film with a polymer layer compared with that of the bare film. This is expected due to the fact that the polymer layer improves the interface and thus the smooth surface reduces the loss of pumping light incident at the air interface with the PQD thin film [31,33,37]. This increase is reduced when the perovskite film is sandwich between two polymer layers (Figure 5a).

Distinctly, the PL decay profile was fitted at the peak position using single-exponential decay. As shown in Figure 5b. A PMMA/PQD and PMMA/PQD/PMMA were revealed decay times of ~17.0 ns and 16.4, respectively, which is longer than that of the PQD (~14.0 ns), which arose because of surface passivation which the passivation of defects in the perovskite top surface [37]. Figure 6 shows the pump-dependent emission from three configurations, through a range of excitation energy densities. The transition from a broad PL spectrum to a narrower ASE feature has occurred at 22.2, 19.3, and, 16.4 μJ/cm^2^ for the configurations PQD, PMMA/CsPbBr_3_, and PMMA/CsPbBr_3_/PMMA, respectively (Figure 7). This improved PL and the subsequent slower emission are the essences of the observed reduction in the ASE threshold. The remaining ASE threshold decrease was instead ascribed to improved waveguiding, which was facilitated by the realization that an almost symmetric PMMA/perovskite/glass and PMMA/perovskite/PMMA/glass waveguide results in higher mode confinement in the perovskite layer concerning the asymmetric perovskite/glass layer.

Figure 8 depicts the ASE intensity dependence as a function of carrier density (*n*). The carrier densities are calculated from the absorbance spectra (Figure 4) and film thickness (estimated from a Dektak 150 stylus profiler). The calculation method has been explained in detail in previously published work [31,47]. The transition from spontaneous emission (SE) to ASE occurs, with a clear onset, at ASE threshold carrier density of ~6.7 × 10^18^ cm^−3^, 2.8 × 10^18^ cm^−3^ and 1.8 × 10^18^ cm^−3^, CsPbBr_3_/glass, PMMA/CsPbBr_3_/glass, and PMMA/CsPbBr_3_/PMMA/glass, respectively. Thus, the PMMA/CsPbBr_3_/PMMA/glass configuration is the best for gain and light amplification applications. This is because, for very high carrier densities, the Coulomb interaction between electrons and holes can induce additional effects. Figure 9 shows ASE stability of the PQD films, before and after PMMA coating under long excitation with over 120 min laser excitation lasts without interruption (for 108,000 excitation shots) by picosecond laser pulses. It is noted that, for the film coated by PMMA, the intensity output does not deviate from the original output until more than 110,000 excitation shots. The improved stability can be ascribed to the incorporation into hydrophobic polymer matrices [45]. By contrast, the ASE, even after 80,000 shots, from the bare sample without coating, can remain at approximately 95% of its original emission intensity. Thus, the ASE photostability of synthesized films is significantly improved after PMMA coating.

For the film with coating, the much stronger and nearly invariant output ASE intensity suggests good optical stability. Such improvement is a result of reduced ASE threshold and increased ASE intensity at a certain pump density, which implies that less heat is generated during operation. As discussed above, it does not only reduce the ASE threshold but also improves the photostability of the films through a simple polymer coating process. This simple technique provides a pathway to improve the photostability of perovskite materials for a sustainable laser. Additionally, CsPbBr_3_ PQDs are verified to be sufficiently stable as optical materials to achieve laser devices. Finally, the stable and continuous laser operation observed here is promising for future applications as it indicates both high photostability and thermal stability (thermal produce by laser pulse) under environmental conditions. Through the coating of a ~100 nm PMMA layer, perovskite films show remarkably enhanced PL and a prolonged decay lifetime. Most importantly, the ASE threshold of the perovskite films is significantly reduced, from 22.2 to 16.4 μJ/cm^2^, with greatly improved light exposure stability. Then, the PMMA polymer layer plays a role that coincides with both surface passivation and symmetric waveguiding have been confirmed. A lower ASE threshold in perovskite films is conducive to stable and sustained output of laser light.

## 5. Conclusions

In conclusion, we have demonstrated that our simple strategy can improve the optical properties of CsPbBr_3_ PQDs by modifying the upper surfaces of the CsPbBr_3_ PQD film or both its upper and lower surfaces. High-quality films were directly synthesized from CsPbBr_3_ PQD powder. The perovskite layer was coated to examine the optical response of the perovskite film after passivation of the surface by PMMA. PMMA acts as a reflector to enhance line narrowing and ASE from perovskite films toward lasing in the PQD structures. These advantages also suggest the great potential of inorganic perovskite films to support stimulated emission. The coating of two perovskite film faces resulted in an ultra-flexible film. The stability of CsPbBr_3_ PQDs films was greatly improved by PMMA coating because of strict isolation from air and moisture in the atmosphere. The ASE thresholds was found of ~16.4 μJ/cm^2^ on flexible PMMA reflectors, which are lower than values reported elsewhere under picosecond laser excitation for bare CsPbBr_3_ PQDs films under picosecond excitation. This work suggests a promising pathway to flexible substrates that may additionally act as the reflector. This work has also confirmed that the PMMA layer plays the roles of both the surface passivation layer and symmetric waveguide. The lowering of the ASE threshold in perovskite films will result in stable, continuous production of perovskite laser light.

## Figures and Tables

**Table 1:** X-ray diffraction (XRD) parameters for various samples.

Sample	FWHM	D	Lattice Strain ε × 10^−3^	Dislocation Density δ × 10^−3^ (nm)^−2^
	(Degrees)	(nm)		
(PMMA/PQDs/PMMA)	1.35	6.0	5.80	27.98
(PMMA/PQDs)	1.22	6.6	5.22	22.69
Pure PQDs	1.40	5.8	5.99	29.87

## Data Availability

The data presented in this study are available on request from the corresponding author.
